# ﻿An updated checklist of *Anopheles* (Diptera, Culicidae) of Colombia with new records and distribution data

**DOI:** 10.3897/zookeys.1231.133711

**Published:** 2025-03-12

**Authors:** Nelson Naranjo-Díaz, Margarita M. Correa

**Affiliations:** 1 Grupo Microbiología Molecular, Escuela de Microbiología, Universidad de Antioquia, Calle 70 No. 52-21, Medellín, Colombia Universidad de Antioquia Medellín Colombia

**Keywords:** *Anopheles*, biodiversity, Colombia, ecological distribution, inventory, malaria vectors

## Abstract

Several species of *Anopheles* mosquitoes (Arthropoda, Insecta, Diptera, Culicidae) are important in public health due to their role in malaria transmission. Of the more than 500 *Anopheles* species worldwide, 47 have been reported in Colombia, but only nine are known to transmit malaria. Taxonomic classification of these mosquitoes is complicated by the existence of species complexes and groups of closely related species that are difficult to distinguish based on morphological characteristics. However, molecular techniques have contributed to resolving taxonomic uncertainties, definition of molecular variants and facilitated the correction of erroneous taxonomic assignments. This study aimed to update the list of *Anopheles* species reported for Colombia. A species checklist was compiled by reviewing catalogs, publications, databases, and unpublished data. Only formally characterized species were included, along with their geographic range and ecological distribution. The final list includes 44 formally characterized *Anopheles* species belonging to five subgenera. The Nyssorhynchus subgenus constituted the largest group with 17 species and the widest distribution, occurring in 18 ecoregions. The Anopheles subgenus was the second largest group with 16 species and occurrences in 16 ecoregions. Sixty-six new presence records were added to the checklist. The updated *Anopheles* checklist, encompassing presence records and ecological distributions, enhances our understanding of *Anopheles* mosquito biodiversity. Furthermore, it contributes to improved public health by providing a foundation for targeted vector control interventions.

## ﻿Introduction

The *Anopheles* genus is highly diverse, with 511 formally recognized species belonging to eight subgenera, including various species complexes. Some of these complexes still contain unnamed members (Harbach 2023). *Anopheles* mosquitoes thrive in a wide range of ecosystems, contributing to their broad geographical distribution. Additionally, some species have adapted to anthropically modified habitats (Hiwat and Bretas 2011; Harbach 2023). The primary importance of this genus lies in its role as a vector of human and animal pathogens, particularly, *Plasmodium* parasites, the causative agents of malaria (WHO 2022). Some *Anopheles* species also transmit *Wuchereriabancrofti*, the nematode that causes filariasis in Africa and Asia (Manguin et al. 2010), and O’nyong-nyong virus, which produces polyarthritis and fever in Africa (Brault et al. 2004).

Most of Colombia has ecological conditions that favor the widespread distribution of *Anopheles* mosquitoes (Olano et al. 2001; IGAC 2002; Hernández-Valencia et al. 2020). In the country, 47 species have been identified (Montoya-Lerma et al. 2011; Gómez et al. 2015), and nine of these are considered malaria vectors. Three species from the Nyssorhynchus subgenus play a significant role in malaria transmission: *Anophelesdarlingi*, *An.nuneztovari*, and *An.albimanus* (Olano et al. 2001; Gutiérrez et al. 2008). *Anophelesdarlingi* is the primary malaria vector in Latin America (Hiwat and Bretas 2011) and is predominantly found in the northwest, east and Amazon regions of Colombia. *Anophelesnuneztovari* is more common in the northwest, northeast and east, while *An.albimanus* is primarily present in coastal areas (Olano et al. 2001; Naranjo-Díaz et al. 2016a, b).

Knowledge of *Anopheles* species in Colombia is based primarily on catalogs published in the mid-20^th^ century (Gast 1943; Barreto-Reyes 1955; Stone et al. 1959; Knight and Stone 1977) and older reports from the former government malaria control program “Servicio Nacional de Erradicación de la Malaria-SEM” (SEM 1957). Subsequent contributions by Carrejo and González (1992) and González and Carrejo (2007, 2009), focusing on the taxonomy and control of medically important insects, with an emphasis on population and taxonomic studies, have significantly improved our understanding of *Anopheles* in Colombia. While the number of reports on *Anopheles* species occurrence and distribution has increased, information remains scattered and, in some cases, inaccessible to the public.

Molecular tools have significantly improved the resolution of taxonomic ambiguities, particularly within species complexes and among closely related species; they have also facilitated the correction of erroneous taxonomic assignments (Brochero et al. 2007; Ruiz-Lopez et al. 2012; Escovar et al. 2014; Gómez et al. 2015). These findings underscore the need for an updated list of species occurrence. A comprehensive checklist of *Anopheles* species in Colombia, incorporating current ecological and geographical data, is essential for advancing medical entomological research and biodiversity assessment.

## ﻿Materials and methods

To update the *Anopheles* species checklist, we reviewed various sources, including mosquito catalogs (Gast 1943; Barreto-Reyes 1955; Knight and Stone 1977; Heinemann and Belkin 1978; Faran 1980; Carrejo and Gonzalez 1992), taxonomic keys (Faran and Linthicum 1981; Gonzalez and Carrejo 2009), and government reports (SEM 1957). Additionally, we obtained data on *Anopheles* species occurrence, including geographical coordinates, from databases (Gaffigan et al. 2014; SIB Database 2020; GBIF 2024), scientific articles, and unpublished new records of specimens collected by members of our research group (named “new occurrence data”). The identities of the new records were previously verified using molecular methods, e.g., COI barcoding or ITS2 analysis (Zapata et al. 2007; Cienfuegos et al. 2011; Gómez et al. 2015) (Suppl. material 1).

To be included in the checklist, an *Anopheles* species was required to have a formal description and validation (Harbach 2023; ITIS 2024). Species variants originally described using only molecular methods were excluded. The checklist includes species listed in alphabetic order. Each entry contains the genus, subgenus, authorship, and year of description; (new occurrence data): This designation is used to indicate that the records are the result of previous work by our research group. Geographical distribution is presented at the level of Colombian administrative departments. An asterisk (*) indicates species occurrences with associated geographical coordinates.

**Figure 1.**
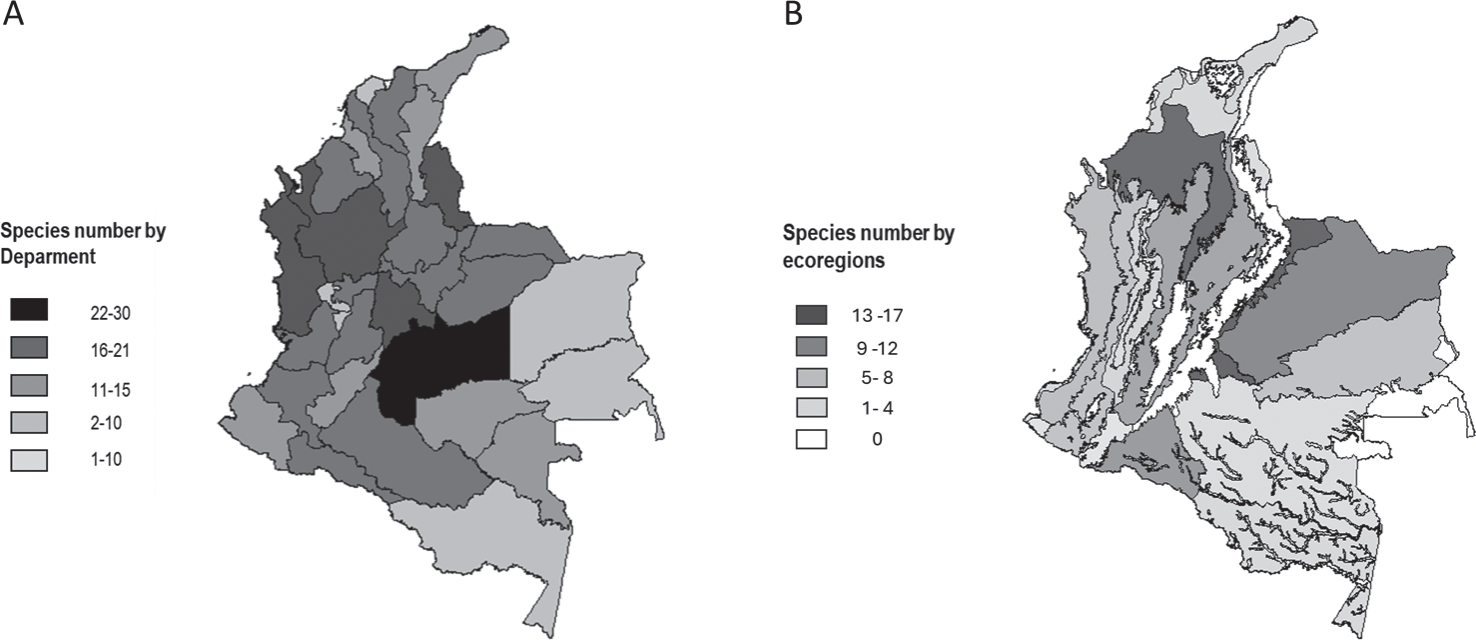
Maps depicting the number of *Anopheles* species reported in Colombia **A** by administrative area (Department) **B** by ecological region.

**Figure 2.**
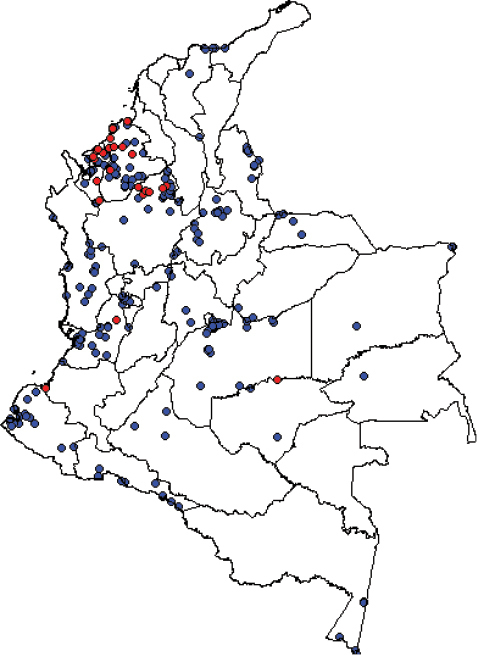
Map indicating the location of species records, according to geographic coordinates. Blue: previously reported records; red: new occurrence data.

Notes provide information on the sources of the data, changes in the taxonomic classification of species, molecular species designations, and new occurrence data, including the municipalities where specimens were collected. The checklist is accompanied by a map illustrating the distribution of *Anopheles* species with associated geographical coordinates (Figs 1, 2, Suppl. material 1).

In addition to the list, a summary table of the *Anopheles* records by administrative departments is included (Table 1), and also, a table of species distribution per ecoregion (WWF 2015) (Table 2), which includes the following: Amazon-Orinoco-Southern Caribbean mangroves, Apure-Villavicencio dry forests, Caquetá moist forests, Catatumbo moist forests, Cauca Valley dry forests, Cauca Valley montane forests, Chocó-Darién moist forests, Cordillera Oriental montane forests, Eastern Cordillera Real montane forests, Guajira-Barranquilla xeric scrub, Llanos, Magdalena Valley montane forests, Magdalena-Urabá moist forests, Napo moist forests, Negro-Branco moist forests, Northwestern Andean montane forests, Sinú Valley dry forests, Solimões–Japurá moist forests, South American Pacific mangroves, Southwest Amazon moist forests, and Western Ecuador moist forests.

**Table 1.** Summary of *Anopheles* records by Colombian administrative departments.

Department	Subgenera	Species	New geographic coordinate registers^*^	Total geographic coordinate registers^£^
Amazonas	3	8	-	11
Antioquia	3	22	40	155
Arauca	2	16	-	6
Atlántico	2	10	-	1
Bolívar	3	17	-	-
Boyacá	3	17	-	4
Caldas	3	17	-	1
Caquetá	4	18	-	6
Casanare	2	16	-	2
Cauca	4	18	1	42
Cesar	2	15	-	-
Chocó	4	23	-	31
Córdoba	2	18	21	80
Cundinamarca	5	23	-	2
Guainía	2	7	-	-
Guaviare	3	13	3	5
Huila	3	13	-	-
La Guajira	2	15	-	4
Magdalena	3	17	-	6
Meta	5	30	-	60
Nariño	4	15	-	35
Norte de Santander	4	24	-	14
Putumayo	3	18	-	36
Quindío	2	7	-	-
Risaralda	2	7	-	3
San Andres y Providencia	1	1	-	-
Santander	2	19	-	33
Sucre	2	13	-	-
Tolima	3	18	-	1
Valle del Cauca	4	19	1	33
Vaupés	5	15	-	-
Vichada	2	10	-	9

* Number of new geographic coordinate records described during this study; ^£^ Total number of geographic coordinate records, including both previous and new occurrence data.

**Table 2.** List of *Anopheles* recorded in Colombia by ecoregion.

Ecoregions*	Species
Amazon-Orinoco-Southern Caribbean mangroves	An. (Ano.) apicimacula, An. (Ano.) punctimacula, *An. (Ano.) pseudopunctipennis^±^*
Apure-Villavicencio dry forests	An. (Ano.) costai, An. (Ano.) apicimacula, *An. (Ano.) pseudopunctipennis^±^*, An. (Ker) bambusicolus, An. (Ker) homunculus, *An. (Ker) pholidotus^±^*, An. (Nys.) albitarsis, An. (Nys.) argyritarsis, *An. (Nys.) benarrochi^±^*, An. (Nys.) braziliensis, *An. (Nys.) darlingi^¥^*, An. (Nys.) marajoara, *An. (Nys.) nuneztovari^¥^*, An. (Nys.) rangeli, An. (Nys.) triannulatus, An. (Ste.) nimbus
Caquetá moist forests	*An. (Nys.) darlingi^¥^*, An. (Nys.) braziliensis
Catatumbo moist forests	An. (Ano.) malefactor, An. (Ano.) neomaculipalpus, An. (Nys.) albitarsis, An. (Nys.) marajoara, *An. (Nys.) nuneztovari^¥^*
Cauca Valley dry forests	*An. (Ano.) calderoni^±^*, *An. (Ano.) punctimacula^±^*, *An. (Ano.) pseudopunctipennis^±^*, *An. (Ker) neivai^±^*, *An. (Nys.) albimanus^¥^*
Cauca Valley montane forests	An. (Ano.) apicimacula, *An. (Ano.) calderoni^±^*, *An. (Ano.) pseudopunctipennis^±^*, *An. (Nys.) albimanus^¥^*, An. (Nys.) albitarsis, *An. (Nys.) nuneztovari^¥^*, An. (Nys.) triannulatus
Chocó-Darién moist forests	An. (Ano.) apicimacula, *An. (Ano.) calderoni^±^*, *An. (Ano.) costai/forattinii*, An. (Ano.) malefactor, *An. (Ano.) punctimacula^±^*, *An. (Ker) neivai^±^*, *An. (Nys.) albimanus^¥^*, *An. (Nys.) darlingi^¥^*, An. (Nys.) eiseni, *An. (Nys.) nuneztovari^¥^*, An. (Nys.) triannulatus
Cordillera Oriental montane forests	An. (Ker) homunculus, *An. (Nys.) darlingi^¥^*, An. (Nys.) rangeli
Eastern Cordillera Real montane forests	An. (Nys.) argyritarsis
Guajira-Barranquilla xeric scrub	An. (Ano.) neomaculipalpus, *An. (Ano.) punctimacula^±^*
Llanos	An. (Ano.) costai, An. (Ano.) peryassui, *An. (Ano.) pseudopunctipennis^±^*, An. (Ano.) shannoni, An. (Ker) bambusicolus, An. (Nys.) albitarsis, An. (Nys.) argyritarsis, An. (Nys.) braziliensis, *An. (Nys.) darlingi^¥^*, An. (Nys.) marajoara, *An. (Nys.) nuneztovari^¥^*, An. (Nys.) oswaldoi, An. (Nys.) rangeli, An. (Nys.) triannulatus
Magdalena Valley montane forests	An. (Ano.) apicimacula, An. (Ano.) neomaculipalpus, *An. (Ano.) pseudopunctipennis^±^*, An. (Nys.) argyritarsis, *An. (Nys.) darlingi^¥^*, *An. (Nys.) nuneztovari^¥^*, An. (Nys.) parvus, An. (Nys.) rangeli, An. (Nys.) triannulatus
Magdalena-Urabá moist forests	An. (Ano.) apicimacula, An. (Ano.) malefactor, An. (Ano.) mattogrossensis, An. (Ano.) neomaculipalpus, An. (Ano.) peryassui, *An. (Ano.) pseudopunctipennis^±^*, *An. (Ano.) punctimacula^±^*, *An. (Ker) neivai^±^*, An. (Lop) squamifemur,*An. (Nys.) albimanus^¥^*, An. (Nys.) albitarsis, An. (Nys.) aquasalis, An. (Nys.) argyritarsis, *An. (Nys.) benarrochi^±^*, An. (Nys.) braziliensis, *An. (Nys.) darlingi^¥^*, An. (Nys.) evansae, An. (Nys.) marajoara, *An. (Nys.) nuneztovari^¥^*, An. (Nys.) oswaldoi, An. (Nys.) rangeli, An. (Nys.) strodei, An. (Nys.) triannulatus, An. (Ste.) nimbus
Napo moist forests	An. (Ano.) costai, An. (Ano.) mattogrossensis, An. (Ano.) neomaculipalpus, *An. (Ano.) punctimacula^±^*, An. (Nys.) albitarsis, An. (Nys.) bellator, An. (Nys.) benarrochi±, An. (Nys.) braziliensis, *An. (Nys.) darlingi^¥^*, An. (Nys.) marajoara, An. (Nys.) oswaldoi, An. (Nys.) rangeli, An. (Nys.) strode, An. (Nys.) triannulatus
Negro-Branco moist forests	An. (Ano.) mattogrossensis, An. (Ano.) peryassui, An. (Nys.) braziliensis, *An. (Nys.) darlingi^¥^*, An. (Nys.) oswaldoi
Northwestern Andean montane forests	*An. (Ano.) calderoni^±^*, *An. (Ano.) pseudopunctipennis^±^*, *An. (Nys.) albimanus^¥^*, An. (Nys.) argyritarsis, *An. (Nys.) darlingi^¥^*, *An. (Nys.) nuneztovari^¥^*, An. (Nys.) triannulatus
Sinú Valley dry forests	*An. (Ano.) punctimacula^±^*
Solimões–Japurá moist forests	An. (Ano.) costai, An. (Ano.) peryassui, *An. (Nys.) darlingi^¥^*, An. (Nys.) dunhami
South American Pacific mangroves	An. (Ano.) apicimacula, *An. (Ano.) calderoni^±^*, *An. (Ker) neivai^±^*, *An. (Nys.) albimanus^¥^*
Southwest Amazon moist forests	*An. (Nys.) darlingi^¥^*
Western Ecuador moist forests	*An. (Ano.) calderoni^±^*, *An. (Nys.) albimanus^¥^*

* Ecoregions according to WWF (2015). Subgenera *Ano*: *Anopheles*, *Ker*: *Kerteszia*, *Lop*: *Lophopodomyia*, *Nys*: *Nyssorhynchus*, *Ste*: *Stethomyia*. ^¥^ Primary malaria vector; ^±^ Local malaria vector.

## ﻿Checklist of *Anopheles* mosquitos in Colombia

### ﻿Order Diptera Linnaeus, 1758

**Infraorder Culicomorpha Hennig, 1948**

**Superfamily Culicoidea Meigen, 1818**

**Family Culicidae Meigen, 1818**

**Subfamily Anophelinae Grassi, 1900**

**Genus *Anopheles* Meigen, 1818**

**Subgenus Anopheles Meigen, 1818**

**Anopheles (Anopheles) apicimacula Dyar & Knab, 1906**

**Distribution.** Antioquia*, Arauca, Bolívar, Boyacá, Caldas, Casanare, Cauca, Cesar, Chocó*, Córdoba*, Cundinamarca, Guaviare, Huila, La Guajira, Magdalena, Meta*, Nariño*, Norte de Santander, Putumayo, Risaralda, Santander*, Sucre, Tolima, Valle del Cauca*.

**Notes.** Reported by Barreto-Reyes (1955), Knight and Stone (1977), Brochero et al. (2005), González and Carrejo (2009), Parra-Henao et al. (2012), Montoya et al. (2017), SIB (2020), Zuleta-Ruiz et al. (2022).

**Anopheles (Anopheles) calderoni Wilkerson, 1991**

**Distribution.** Antioquia, Caldas, Chocó*, Huila, La Guajira, Magdalena, Nariño*, Norte de Santander, Quindio, Risaralda*, Tolima, Valle del Cauca*.

**Notes.** Local malaria vector. Reported by González and Carrejo (2009), González et al. (2010), Lucumi-Aragón et al. (2011), Orjuela et al. (2015), Montoya et al. (2017), Galeano-Castañeda et al. (2019), SIB Database (2020).

**Anopheles (Anopheles) costai Da Fonseca & Ramos, 1939**

**Distribution.** Amazonas*, Antioquia, Arauca, Bolívar, Boyacá, Caldas, Caquetá, Cesar, Chocó, Córdoba, Cundinamarca, Guainía, Guaviare, Huila, Meta*, Nariño, Putumayo*, Santander, Sucre, Valle del Cauca, Vaupés.

**Notes.** Reported by González and Carrejo (2009), Gutiérrez et al. (2009), Ahumada et al. (2013), Orjuela et al. (2013), SIB (2020). *Anophelescostai* was resurrected from synonymy with *Anophelesmediopunctatus* (Sallum et al. 1999), and it was previously erroneously reported in Colombia as *An.mediopunctatus*, as pointed out by Sallum et al. (1999) and Quiñones et al. (2001).

**Anopheles (Anopheles) eiseni Coquillett, 1902**

**Distribution.** Antioquia, Boyacá, Caldas, Casanare, Cauca, Chocó*, Cundinamarca, Huila, La Guajira, Magdalena, Meta, Nariño, Norte de Santander, Quindío, Risaralda, Santander, Tolima, Valle del Cauca*.

**Notes.** Reported by Barreto-Reyes (1955), Knight and Stone (1977), SIB (2020).

**Anopheles (Anopheles) fluminensis Root, 1927**

**Distribution.** Norte de Santander.

**Notes.** Reported by González and Carrejo (2009).

**Anopheles (Anopheles) forattinii Wilkerson & Sallum, 1999**

**Distribution.** Meta, Vaupés.

**Notes.** Reported by Wilkerson and Sallum (1999). It was indicated that *An.forattinii* was previously reported in Colombia as *An.mediopunctatus* (Sallum et al. 1999; Quiñones et al. 2001).

**Anopheles (Anopheles) malefactor Dyar & Knab, 1907**

**Distribution.** Antioquia*, Chocó*, Córdoba*, Meta, Norte de Santander*.

**Notes.** Reported by Wilkerson (1990), Álvarez et al. (2018), SIB Database (2020). New occurrence data from Monteria municipality, Córdoba Department.

**Anopheles (Anopheles) mattogrossensis Lutz & Neiva, 1911**

**Distribution.** Amazonas*, Arauca, Caquetá*, Cauca, Guainía, Guaviare, Meta, Norte de Santander, Putumayo, Vaupés, Vichada*.

**Notes.** Reported by Barreto-Reyes (1955), Knight and Stone (1977), Brochero et al. (2006), Orjuela et al. (2013), Brochero and Conn (2015), Álvarez et al. (2018), Prado et al. (2019).

**Anopheles (Anopheles) neomaculipalpus Curry, 1931**

**Distribution.** Amazonas, Antioquia*, Arauca, Atlántico, Bolívar, Boyacá, Caldas, Casanare, Caquetá, Cauca, Cesar, Chocó, Córdoba*, Cundinamarca, Guaviare, Huila, La Guajira*, Magdalena, Meta, Nariño, Norte de Santander*, Putumayo*, Santander*, Sucre, Tolima, Valle del Cauca, Vaupés.

**Notes.** Reported by Barreto-Reyes (1955), Knight and Stone (1977), Brochero et al. (2006), Parra-Henao and Alarcón (2008), González and Carrejo (2009), Parra-Henao et al. (2012), Orjuela et al. (2013), Álvarez et al. (2018). New occurrence data from Caceres Municipality, Antioquia Department, and Monteria and Valencia municipalities, Córdoba Department.

**Anopheles (Anopheles) peryassui Dyar & Knab, 1908**

**Distribution.** Amazonas*, Antioquia*, Caldas, Caquetá, Casanare, Cundinamarca, Guainía, Guaviare, Meta*, Putumayo, Santander*, Sucre, Vaupés, Vichada*.

**Notes.** Reported by Barreto-Reyes (1955), Knight and Stone (1977), González and Carrejo (2009), Brochero and Conn (2015), Álvarez et al. (2018), SIB (2020).

**Anopheles (Anopheles) pseudopunctipennis Theobald, 1901**

**Distribution.** Antioquia*, Arauca, Atlántico, Bolívar, Boyacá, Caldas, Caquetá, Casanare, Cauca, Cesar, Chocó, Córdoba*, Cundinamarca, Guaviare, Huila, La Guajira, Magdalena*, Meta*, Nariño, Norte de Santander, Putumayo, Quindío, Risaralda, Santander*, Sucre, Tolima, Valle del Cauca*.

**Notes.** Local malaria vector. Reported by Barreto-Reyes (1955), Knight and Stone (1977), Brochero et al. (2006), Parra-Henao and Alarcón (2008), González and Carrejo (2009), Parra-Henao et al. (2012), Naranjo-Díaz et al. (2013), Montoya et al. (2017), SIB (2020). New occurrence data from Apartadó, Arboletes, Cáceres, Necoclí, San Juan de Urabá and Tarazá Municipalities in Antioquia Department, and Canalete Municipality in Córdoba Department.

**Anopheles (Anopheles) punctimacula Dyar & Knab, 1906**

**Distribution.** Antioquia*, Arauca, Atlántico, Bolívar, Boyacá*, Caldas, Caquetá, Casanare, Cauca, Cesar, Chocó*, Córdoba*, Cundinamarca, Guaviare, Huila, La Guajira*, Magdalena*, Meta, Nariño*, Norte de Santander, Putumayo*, Quindío, Risaralda, Santander, Sucre, Tolima, Valle del Cauca*, Vaupés, Vichada.

**Notes.** Local malaria vector. Reported by Barreto-Reyes (1955), Knight and Stone (1977), González and Carrejo (2009), Gutiérrez et al. (2009), Orjuela et al. (2013), Naranjo-Díaz et al. (2013, 2014), Montoya et al. (2017), Álvarez et al. (2018), Galeano-Castañeda et al. (2019), SIB (2020). New occurrence data from Apartadó, Caucasia and Necoclí Municipalities in Antioquia Department, and Canalete Municipality in Córdoba Department.

**Anopheles (Anopheles) shannoni Davis, 1931**

**Distribution.** Vaupés, Vichada*.

**Notes.** Reported by González and Carrejo (2009), Brochero and Conn (2015).

**Anopheles (Anopheles) vestitipennis Dyar & Knab, 1906**

**Distribution.** Cesar, Valle del Cauca.

**Notes.** Reported by Barreto-Reyes (1955), Knight and Stone (1977).

#### ﻿Subgenus Kerteszia Theobald, 1905

**Anopheles (Kerteszia) bambusicolus Komp, 1937**

**Distribution.** Caquetá, Meta*.

**Notes.** Reported by Gast (1943), Barreto-Reyes (1955), Knight and Stone (1977), SIB Database (2020).

**Anopheles (Kerteszia) bellator Dyar & Knab, 1906**

**Distribution.** Putumayo*.

**Notes.** Reported by Barreto-Reyes (1955), Knight and Stone (1977), SIB Database (2020).

**Anopheles (Kerteszia) boliviensis (Theobald, 1905)**

**Distribution.** Caldas, Cauca, Chocó, Cundinamarca, Huila, Meta, Nariño, Tolima.

**Notes.** Reported by Barreto-Reyes (1955), Knight and Stone (1977), González and Carrejo (2009).

**Anopheles (Kerteszia) homunculus Komp, 1937**

**Distribution.** Boyacá, Cauca, Chocó, Cundinamarca, Meta*, Norte de Santander, Tolima.

**Notes.** Reported by Barreto-Reyes (1955), Knight and Stone (1977), González and Carrejo (2009), SIB Database (2020).

**Anopheles (Kerteszia) neivai Howard, Dyar & Knab, 1913**

**Distribution.** Antioquia, Bolívar, Boyacá, Cauca, Chocó*, Cundinamarca, Nariño*, Norte de Santander, Tolima, Valle del Cauca*, Vaupés.

**Notes.** Local malaria vector. Reported by Barreto-Reyes (1955), Knight and Stone (1977), Solarte et al. (1996), Gutierréz et al. (2008, 2009), González and Carrejo (2009), Naranjo-Díaz et al. (2014, 2023), SIB (2020).

**Anopheles (Kerteszia) pholidotus Zavortink, 1973**

**Distribution.** Caquetá, Cundinamarca, Magdalena, Meta*, Norte de Santander, Putumayo*, Tolima*, Valle del Cauca.

**Notes.** Local malaria vector. Reported by González and Carrejo (2009), Escovar et al. (2014), SIB (2020). Previously reported as *Anopheleslepidotus* (Escovar et al. 2014), a species that is not present in the country.

#### ﻿Subgenus Lophopodomyia Antunes, 1937

**Anopheles (Lophopodomyia) gilesi (Peryassú, 1908)**

**Distribution.** Meta.

**Notes.** Reported by Barreto-Reyes (1955), Knight and Stone (1977).

**Anopheles (Lophopodomyia) oiketorakras Osorno-Mesa, 1947**

**Distribution.** Cundinamarca, Nariño.

**Notes.** Reported by Barreto-Reyes (1955), Knight and Stone (1977).

**Anopheles (Lophopodomyia) squamifemur Antunes, 1937**

**Distribution.** Antioquia*, Cauca, Chocó, Norte de Santander, Valle del Cauca. Vaupés.

**Notes.** Reported by Barreto-Reyes (1955), Knight and Stone (1977). New occurrence data from Cáceres Municipality in Antioquia Department.

#### ﻿Subgenus Nyssorhynchus Blanchard, 1902

**Anopheles (Nyssorhynchus) albimanus Wiedemann, 1820**

**Distribution.** Antioquia*, Atlántico, Bolívar, Cauca*, Cesar, Chocó*, Córdoba*, La Guajira, Magdalena, Nariño*, Risaralda*, Sucre, San Andrés y Providencia, Valle del Cauca*.

**Notes.** Primary malaria vector. Reported by Barreto-Reyes (1955), Knight and Stone (1977), Faran (1980), Gutierrez et al. (Gutiérrez et al. 2008, 2009), Calle et al. (2008), González and Carrejo (2009), Naranjo-Díaz et al. (2013, 2014, 2023), Montoya et al. (2017), Galeano-Castañeda et al. (2019), Altamiranda-Saavedra et al. (2023), SIB (2020). New occurrence data from Apartadó, Mutatá, Necoclí, San Juan de Urabá and Tarazá Municipalities in Antioquia Department, Guapi Municipality in Cauca Department, Canalete, Monteria and Puerto Escondido Municipalities in Córdoba Department, and El Zarzal Municipality in Valle del Cauca.

**Anopheles (Nyssorhynchus) albitarsis Lynch Arribálzaga, 1878**

**Distribution.** Antioquia*, Meta*, Norte de Santander*, Putumayo*, Vichada*.

**Notes.** Reported by Calle et al. (2008), Gutiérrez et al. (2009), González and Carrejo (2009), Jiménez et al. (2012), Ahumada et al. (2013), Orjuela et al. (2013), Montoya et al. (2017), Galeano-Castañeda et al. (2019), SIB (2020). In Colombia, *Anophelesalbitarsis* was previously reported as *An.albitarsis* s.l. or *An.marajoara*, both are part of the Albitaris Complex, which potentially comprises at least ten species, only five have been formally described (Bourke et al. 2021). Only the molecular variants *An.albitarsis* F and *An.albitarsis* I are reported in the country, *An.marajoara* could not be confirmed (Ruiz-Lopez et al. 2012). New occurrence data from Arboletes and Caucasia Municipalities in Antioquia Department, Moñitos and San Antero Municipalities in Córdoba Department.

**Anopheles (Nyssorhynchus) aquasalis Curry, *1932***

**Distribution.** Atlántico, Bolívar, Chocó, Córdoba*, La Guajira*, Magdalena

**Notes.** Reported by Barreto-Reyes (1955), Knight and Stone (1977), Calle et al. (2008). New occurrence data from San Antero Municipality in Córdoba Department.

**Anopheles (Nyssorhynchus) argyritarsis Robineau-Desvoidy, 1827**

**Distribution.** Antioquia*, Arauca, Atlántico, Bolívar, Boyacá, Caldas, Caquetá, Casanare, Cauca, Cesar, Chocó, Cordobá*, Cundinamarca, Huila, La Guajira, Magdalena, Meta*, Nariño*, Norte de Santander, Putumayo, Quindío, Risaralda, Santander*, Tolima, Valle del Cauca, Vaupés, Vichada*.

**Notes.** Reported by Barreto-Reyes (1955), Knight and Stone (1977), González and Carrejo (2009), Jiménez et al. (2012), Parra-Henao et al. (2012)Naranjo-Díaz et al. (2013), SIB (2020). New occurrence data from Cáceres Municipality in Antioquia Department.

**Anopheles (Nyssorhynchus) benarrochi Gabaldón, Cova García & Lopez, 1941**

**Distribution.** Meta*, Putumayo*, Santander*.

**Notes.** Local malaria vector. Reported by Barreto-Reyes (1955), Knight and Stone (1977), Quiñones et al. (2001), Calle et al. (2008), González and Carrejo (2009), Parra-Henao et al. (2012), Orjuela et al. (2013). In Colombia, a molecular variant denominated *An.benarrochi* B was reported, distributed in the south of the country (Ruiz et al. 2005).

**Anopheles (Nyssorhynchus) braziliensis Chagas, 1907**

**Distribution.** Amazonas, Antioquia*, Arauca, Bolívar, Boyacá, Caldas, Caquetá, Casanare, Cesar, Chocó, Córdoba, Cundinamarca, Guainía, Guaviare*, Huila, La Guajira, Magdalena, Meta*, Norte de Santander, Putumayo*, Santander, Tolima, Valle del Cauca, Vaupés, Vichada*.

**Notes.** Reported by Barreto-Reyes (1955), Brochero et al. (2005), Calle et al. (2008), González and Carrejo (2009), Naranjo-Díaz et al. (2013, 2023), Jiménez et al. (2012, 2014), Ahumada et al. (2013), SIB (2020). New occurrence data from Arboletes, Cáceres, Caucasia and Tarazá Municipalities in Antioquia Department, San Jose del Guaviare Municipality in Guaviare Department.

**Anopheles (Nyssorhynchus) darlingi Root, 1926**

**Distribution.** Amazonas*, Antioquia*, Arauca, Bolívar, Boyacá, Caldas, Caquetá, Casanare, Cesar, Chocó*, Córdoba*, Cundinamarca, Guainía, Guaviare*, La Guajira, Magdalena, Meta*, Norte de Santander, Putumayo*, Santander, Sucre, Vaupés, Vichada*.

**Notes.** Primary malaria vector. Reported by Barreto-Reyes (1955), Knight and Stone (1977), Brochero et al. (2005), Calle et al. (2008), González and Carrejo (2009), Jiménez et al. (2012, 2014), Ahumada et al. (2013), Orjuela et al. (2013), Naranjo-Díaz et al. (2013, 2014, 2016a, 2019), Montoya et al. (2017), Pacheco et al. (2017), Galeano-Castañeda et al. (2019), SIB (2020). New occurrence data from Apartadó, Cáceres, Caucasia and Necoclí Municipalities in Antioquia Department, San Carlos and Valencia Municipalities in Córdoba Department, San Jose del Guaviare Municipality in Guaviare Department.

**Anopheles (Nyssorhynchus) dunhami Causey, 1945**

**Distribution.** Amazonas*.

**Notes.** Reported by Ruiz et al. (2010).

**Anopheles (Nyssorhynchus) evansae Brèthes, 1926**

**Distribution.** Córdoba*.

**Notes.** Reported by Knight and Stone (1977), Parra-Henao and Alarcon (2008).

**Anopheles (Nyssorhynchus) marajoara Galvão & Damasceno, 1942**

**Distribution.** Antioquia*, Arauca, Atlántico, Bolívar, Boyacá, Caldas, Caquetá*, Casanare, Cauca, Cesar, Chocó, Córdoba, Cundinamarca, Guaviare, Huila, La Guajira, Magdalena*, Meta*, Norte de Santander*, Putumayo, Santander, Sucre, Tolima, Vaupés, Vichada*.

**Notes.** Reported by Knight and Stone (1977), González and Carrejo (2009), Brochero et al. (2010), Jiménez et al. (2012).

**Anopheles (Nyssorhynchus) nuneztovari Gabaldón, 1940**

**Distribution.** Antioquia*, Arauca*, Bolívar, Boyacá, Caldas, Caquetá, Casanare*, Cauca, Cesar, Chocó*, Córdoba*, Cundinamarca, Huila, Magdalena, Meta, Norte de Santander*, Putumayo, Santander*, Sucre, Tolima, Valle del Cauca*.

**Notes.** Primary malaria vector. Reported by Barreto-Reyes (1955), Knight and Stone (1977), Calle et al. (2002, 2008), Brochero et al. (2006), Fajardo et al. (2008), Parra-Henao and Alarcón (2008), González and Carrejo (2009), Fonseca-González et al. (2009), Ruiz et al. (2010), Parra-Henao et al. (2012), Naranjo-Díaz et al. (2013, 2014, 2016b, 2023), Montoya et al. (2017), Galeano-Castañeda et al. (2019), Altamiranda-Saavedra et al. (2023), SIB (2020). New occurrence data from Apartadó, Caucasia, Necoclí and Tarazá Municipalities in Antioquia Department, Monteria and Moñitos Municipalities in Córdoba Department.

**Anopheles (Nyssorhynchus) oswaldoi Peryassú, 1922**

**Distribution.** Amazonas, Antioquia*, Arauca, Atlántico, Bolívar, Boyacá, Caldas, Caquetá, Casanare, Cauca, Cesar, Chocó, Córdoba, Cundinamarca, Guainía, Guaviare*, Magdalena, Meta, Nariño, Norte de Santander, Putumayo*, Santander*, Sucre, Tolima, Valle del Cauca, Vaupés, Vichada*.

**Notes.** Reported by Barreto-Reyes (1955), González and Carrejo (2009), Ruiz et al. (2010), Parra-Henao et al. (2012), Jiménez et al. (2012, 2014), Orjuela et al. (2013). New occurrence data from Caucasia Municipality in Antioquia Department, San Jose del Guaviare Municipality in Guaviare Department.

**Anopheles (Nyssorhynchus) parvus Chagas, 1907**

**Distribution.** Arauca, Casanare, Meta, Santander*, Vichada.

**Notes.** Reported by Barreto-Reyes (1955), Knight and Stone (1977), Parra-Henao et al. (2012), SIB (2020).

**Anopheles (Nyssorhynchus) rangeli Gabaldón, Cova García & Lopez, 1940**

**Distribution.** Antioquia*, Arauca*, Bolívar, Boyacá*, Caldas*, Caquetá*, Casanare, Cauca, Cesar, Chocó, Córdoba*, Cundinamarca*, Guainía, Guaviare*, La Guajira, Magdalena, Meta*, Nariño, Norte de Santander, Putumayo*, Santander*, Tolima, Valle del Cauca.

**Notes.** Reported by Barreto-Reyes (1955), Knight and Stone (1977), Brochero et al. (2005, 2006), Calle et al. (2008), González and Carrejo (2009), Jiménez et al. (2012), Parra-Henao et al. (2012), Orjuela et al. (2013), SIB (2020). New occurrence data from Arboletes and Caucasia Municipalities in Antioquia Department, Moñitos Municipality in Córdoba Department.

**Anopheles (Nyssorhynchus) strodei Root, 1926**

**Distribution.** Antioquia, Arauca*, Bolívar, Boyacá, Caquetá, Casanare, Chocó, Córdoba, Cundinamarca, La Guajira, Meta*, Norte de Santander, Putumayo*, Santander*, Valle del Cauca.

**Notes.** Reported by Barreto-Reyes (1955), Calle et al. (2008), González and Carrejo (2009), Parra-Henao et al. (2012), SIB Database (2020).

**Anopheles (Nyssorhynchus) triannulatus Neiva & Pinto, 1922**

**Distribution.** Amazonas, Antioquia*, Arauca, Atlántico, Bolívar, Boyacá, Caldas, Caquetá, Casanare, Cauca, Cesar, Chocó*, Córdoba*, Cundinamarca, Guaviare, Huila, La Guajira, Magdalena*, Meta*, Nariño, Norte de Santander, Putumayo*, Quindío, Santander*, Sucre, Tolima, Valle del Cauca.

**Notes.** Reported by Barreto-Reyes (1955), Knight and Stone (1977), Brochero et al. (2006), Calle et al. (2008), González and Carrejo (2009), Parra-Henao et al. (2012), Ahumada et al. (2013), Naranjo-Díaz et al. (2013, 2023), Orjuela et al. (2013), Montoya et al. (2017), Atencia-Pineda et al. (2018), SIB (2020). New occurrence data from Apartadó, Arboletes, Caucasia, Necoclí, and San Juan de Urabá Municipalities in Antioquia Department, Canalete, Monteria, Moñitos, San Antero, and Valencia Municipalities in Córdoba Department.

**Anopheles (Nyssorhynchus) trinkae Faran, 1979**

**Distribution.** Meta*.

**Notes.** Reported by González and Carrejo (2009), SIB Database (2020).

#### ﻿Subgenus Stethomyia Theobald, 1902

**Anopheles (Stethomyia) kompi Edwards, 1930**

**Distribution.** Caquetá.

**Notes.** Reported by Barreto-Reyes (1955), Knight and Stone (1977).

**Anopheles (Stethomyia) nimbus (Theobald, 1902)**

**Distribution.** Cundinamarca, Guaviare, Meta, Vaupés, Valle del Cauca.

**Notes.** Reported by Barreto-Reyes (1955), Knight and Stone (1977), González and Carrejo (2009).

**Anopheles (Stethomyia) thomasi Shannon, 1933**

**Notes.** Reported by Barreto-Reyes (1955), Knight and Stone (1977).

## ﻿Discussion

The genus *Anopheles* contains eight subgenera, five of which are present in the Neotropical region (*Anopheles*, *Kerteszia*, *Lophopodomyia*, *Nyssorhynchus*, and *Stethomyia*; Harbach 2023). All five subgenera are present in Colombia. The current checklist only includes formally described species and excludes molecularly identified variants. Therefore, a total of 44 species are listed as present in Colombia. Species of the subgenera *Nyssorhynchus* and *Anopheles* are the most widely distributed in the country, occurring in 32 and 31 Colombian departments, respectively. Nyssorhynchus is the subgenus with the highest species richness, comprising 17 species. *Stethomyia* has a limited distribution, being found in only three departments. *Stethomyia* and *Lophopodomyia* subgenera are represented by three species each. The highest species richness was detected in Meta Department, with a total of 30 species, followed by Norte de Santander with 24. San Andres and Providencia Department exhibited the lowest species richness, with only the main Colombian malaria vector, *An.albimanus*, being recorded in this insular region (Table 1) (Fig. 1).

It is well known that factors such as temperature, rainfall, and humidity affect the geographical distribution of *Anopheles* species (Abiodun et al. 2016). Colombia exhibits a wide variety of ecological conditions, and 34 ecoregions are described (WWF 2015). The current checklist reports the presence of *Anopheles* species in 21 of those ecoregions (Table 2). The Magdalena-Urabá moist forests ecoregion exhibits the highest richness with 23 species, dominated by the species of the subgenera *Nyssorhynchus* and *Anopheles*, 14 and 7 species, respectively. The characteristics of this ecoregion include consistently high temperatures exceeding 28 °C and average annual rainfall within the range of 2,000–4,000 millimeters (WWF 2015). This ecoregion forms part of the important Tumbes-Chocó-Magdalena biodiversity hotspot, which spans southern Panamá to northern Perú and encompasses coastal and lowland areas of the Magdalena River basin and the Urabá region in northwest Colombia. The diverse ecological requirements of *Anopheles* species, which thrive on a variety of landscapes ranging from mosaic tropical rainforests to wetlands, mangrove swamps, and coastal plains, contribute to the high species richness detected in these Colombian regions.

Several members of the genus *Anopheles* are important in public health due to their role as malaria vectors. In Colombia, the annual number of malaria cases has exceeded 70,000 in recent years (INS 2021, 2022, 2023). Of the nine *Anopheles* species implicated in malaria transmission (Olano et al. 2001; Gutiérrez et al. 2008; Orjuela et al. 2013; Naranjo-Díaz et al. 2013), at least one malaria vector species was registered in each of the 21 ecoregions with occurrence data, except for the Eastern Cordillera Real montane forests (Table 1). As the Magdalena-Urabá moist forests ecoregion exhibited the highest species richness, it is not surprising that it also contained most of the malaria vectors, including the three primary vectors (*An.albimanus*, *An.darlingi*, and *An.nuneztovari*) and four local vectors (*An.pseudopunctipennis*, *An.punctimacula*, *An.neivai*, and *An.benarrochi*). The Chocó-Darién moist forests ecoregion was second in the number of vectors present, including the three primary vectors and three local vectors (*An.calderoni*, *An.punctimacula*, and *An.neivai*). These two ecoregions encompass the most important Colombian malaria regions, the Urabá, Bajo Cauca, Alto Sinú, and Pacific regions, which report the highest annual number of malaria cases (INS 2023).

Several factors contribute to the discrepancies between previous catalogs and the current checklist. For example, *An.mediopunctatus* may be restricted to Brazil, and in Colombia, it was likely misidentified as *An.costai* or *An.forattinii* (Sallum et al. 1999; Quiñones et al. 2001). Additionally, *Anophelescruzii*, although reported from Costa Rica to Argentina, has not been confirmed in Colombia (Wilkerson and Peyton 1991). Furthermore, *Anophelesvargasi* has been registered in neighboring Venezuela (Del Ventura et al. 2013), but there is no evidence of its presence in Colombia (Osorno-Mesa 1947). Mistaken taxonomic assignments, often due to the existence of cryptic species, have led to erroneous reports. For instance, *An.pholidotus* was previously reported as *An.lepidotus* (Escovar et al. 2014).

## ﻿Conclusions

In recent decades, numerous studies and surveys conducted in the country have provided valuable data on the presence and distribution of *Anopheles* species. The current checklist attempts to compile the available information. The current list includes 44 formally characterized *Anopheles* species from five subgenera, with the subgenera *Nyssorhynchus* and *Anopheles* being the largest and most widely distributed species groups. Information for 66 new occurrence data is also provided. The incorporation of presence records and ecological distributions is essential for accurately estimating the *Anopheles* species diversity and assessing the malaria vectors. This is fundamental for the design and implementation of effective control interventions.

An interesting finding is that most of the new species records are concentrated in specific regions, likely reflecting research interest in the most malaria-endemic regions of Colombia. It is noteworthy that after 47 species had been recorded in the country, the current checklist only includes 44 species. For an *Anopheles* species to be included in the list, it was required to have a formal description and validation; species variants originally described using only molecular methods were excluded. Among the factors contributing to the discrepancies between previous catalogs and the current checklist are the existence of problematic species, or in some cases damaged specimens, which can lead to misidentifications; also, mistaken taxonomic assignments can occur due to the existence of cryptic species. In addition, for some species, there was no evidence of their presence in Colombia. Finally, while the use of molecular techniques has helped to clarify the taxonomic status of several problematic species, this has led to an increase in the number of molecular variants reported. However, it has also facilitated the correction of erroneous taxonomic assignments, which in turn, may lead to a decrease in the number of species formally described.

## ﻿Additional information

### Author ORCIDs

Margarita M. Correa https://orcid.org/0000-0003-2419-7269

### Data availability

The dataset containing the information on *Anopheles* species occurrence in Colombia is available in: https://doi.org/10.5281/zenodo.13527884. (Data will be available upon publication).

## Supplementary materials

Supplementary material 1 Data on *Anopheles* species occurrence in Colombia Data type xlsx Explanation note Geographic coordinates are shown in decimal degrees and were taken from database portals, scientific articles, and the research group unpublished new occurrence data. https://binary.pensoft.net/file/1278878This dataset is made available under the Open Database License (http://opendatacommons.org/licenses/odbl/1.0/). The Open Database License (ODbL) is a license agreement intended to allow users to freely share, modify, and use this Dataset while maintaining this same freedom for others, provided that the original source and author(s) are credited.Nelson Naranjo-Díaz, Margarita M. Correa
